# Overcrowding in the Medical Profession

**Published:** 1904-09-24

**Authors:** 


### Overcrowding in the Medical Profession.

The reports of the American Consuls at Chemnitz and Frankfort on overcrowding of the medical profession in Germany will be read with interest by every medical man in the United Kingdom. Mr. Monaghan, writing from Chemnitz, says that the overcrowding in the medical profession in Germany is yearly growing worse, yet more and more students crowd into the medical schools. During the last two years the number of practitioners has increased 4 per cent. There are now 29,200 medical practitioners in the country, or twice the number found in 1876. In the larger cities there is a doctor for every 800 inhabitants, while in Berlin nearly half the doctors have a taxable income of less than £150; of these 27 per cent. have incomes ranging between £45 and £150, 13 per cent. have an uncertain income, and 5 per cent. no taxable income at all. In the legal profession, on the other hand, 80 per cent. have an income of over £500 a year. In Saxony the number of doctors has nearly doubled in the last 15 years. The Consul at Frankfort says that the German Association of Physicians is trying to find employment for its members abroad to prevent the overcrowding at home. Hence an information bureau has been established at Hamburg, and a circular has been sent to German Consular officers by the Berlin Foreign Office desiring them to let the bureau know whenever a chance occurs abroad for a German doctor. Now, in simple numbers the German figures about the members of the medical profession compare favourably with those of our own country. The "Medical Register" for 1904 states that the total number of persons on the English Register was 40,572; on the Scotch Register, 15,553; and on the Irish Register, 9,921. The average numbers removed from the register for all causes was 807, and the average number added was 1,305, the average for five years being taken in each case.

The wastage of the profession in England, therefore, is so amply supplied that for each name removed nearly two are added. Yet, although the competition is keen, no one looking at the question with an unbiassed mind would venture to say that the medical profession was overstocked in this country. There appear to be several reasons why Germany, with a smaller total number of medical men, should be complaining of overcrowding when we, with a larger number, have not yet felt the pinch acutely. The most important reason is that to which the German Association of Physicians has very properly drawn attention. Our colonies and our great Indian Empire absorb a large number of medical men annually. Some go with an official position in the Indian Medical or Colonial Medical Service, but a very large number emigrate to form colonists in the same manner as is done by their unqualified brothers and cousins, simply from the love of a less conventional life than is possible here or because they think themselves better suited for a new country. In either case the men who thus go abroad do not enter into competition with those who remain at home. The majority become citizens in the country of their adoption and often attain deservedly to a high position led thereto by their superior education, by their greater energy and by the better knowledge of mankind vouchsafed by the practice of their profession. Those who return do so either on a pension or, as more rarely happens, in the enjoyment of a hardly-earned competency. The different methods of conducting the medical profession at home and abroad are also to our advantage and lead us to feel overcrowding rather less than our neighbours. On the Continent we believe that each man is for himself, and that partnerships, if not wholly unknown, are, at any rate, much less common than in England. Partly from this and partly from other causes the fair-wage system does not hold sway abroad. Neither in Germany nor in France (except in Berlin and in Paris) is it the custom to ask the large fees to which people of the English-speaking countries are gradually becoming accustomed. Money, until lately, has been scarce and the standard of living has been simpler. But as the necessaries of life become dearer and the general idea of comfort becomes higher medical men will be obliged to increase their fees. As soon as it becomes an acknowledged fact that a livelihood cannot be made by the practice of medicine as a profession the overcrowding will cease, and the time will then have arrived for a proper increase in the scale of fees. Meanwhile, the flocking of young men into our profession is not an unmixed evil for it makes still further for the formation of an upper middle [illegible] professional class whose conservative principles ha[illegible] done so much of late years to steady English pub[illegible] opinion.

